# Higher Cytopathic Effects of a Zika Virus Brazilian Isolate from Bahia Compared to a Canadian-Imported Thai Strain

**DOI:** 10.3390/v10020053

**Published:** 2018-01-27

**Authors:** Sergio P. Alpuche-Lazcano, Craig R. McCullogh, Olivier Del Corpo, Elodie Rance, Robert J. Scarborough, Andrew J. Mouland, Selena M. Sagan, Mauro M. Teixeira, Anne Gatignol

**Affiliations:** 1 Virus-Cell Interactions Laboratory, Lady Davis Institute for Medical Research, H3T 1E2, Montréal, QC, Canada; sergio.alpuche@mail.mcgill.ca (S.P.A.-L.); craig.mccullogh@mail.mcgill.ca (C.R.M.); olivier.delcorpo@mail.mcgill.ca (O.D.C.); elodie.rance@mail.mcgill.ca (E.R.); robert.scarborough@mail.mcgill.ca (R.J.S.); 2 RNA Trafficking Laboratory, Lady Davis Institute for Medical Research, H3T 1E2, Montréal, QC, Canada; andrew.mouland@mcgill.ca; 3 Department of Medicine, Division of Experimental Medicine, McGill University, H4A 3J1, Montréal, QC, Canada; 4 Department of Microbiology and Immunology, McGill University, H3A 2B4, Montréal, QC, Canada; selena.sagan@mcgill.ca; 5 Department of Biochemistry, McGill University, H3A 1A3, Montréal, QC, Canada; 6 Departamento de Bioquimica e Imunologia do Instituto de Ciencias Biologicas, Universidade Federal de Minas Gerais, 31270-901, Belo Horizonte, MG, Brazil; mmtex.ufmg@gmail.com

**Keywords:** Zika virus, predicted protein structure, qRT-PCR, cytopathicity, viral titer

## Abstract

Zika virus (ZIKV) is an emerging pathogen from the *Flaviviridae* family. It represents a significant threat to global health due to its neurological and fetal pathogenesis (including microcephaly and congenital malformations), and its rapid dissemination across Latin America in recent years. The virus has spread from Africa to Asia, the Pacific islands and the Americas with limited knowledge about the pathogenesis associated with infection in recent years. Herein, we compared the ability of the Canadian-imported Thai strain PLCal_ZV and the Brazilian isolate HS-2015-BA-01 from Bahia to produce infectious ZIKV particles and cytopathic effects in a cell proliferation assay. We also compared the intracellular viral RNA accumulation of the two strains by quantitative RT-PCR (reverse transcription polymerase chain reaction) analyses. Our observations show that HS-2015-BA-01 is more cytopathic than PLCal_ZV in proliferation assays in Vero, Human Embryonic Kidney HEK 293T and neuroblastoma SH-SY5Y cells. Quantitative RT-PCR shows that the level of viral RNA is higher with HS-2015-BA-01 than with PLCal_ZV in two cell lines, but similar in a neuroblastoma cell line. The two strains have 13 amino acids polymorphisms and we analyzed their predicted protein secondary structure. The increased cytopathicity and RNA accumulation of the Brazilian ZIKV isolate compared to the Thai isolate could contribute to the increased pathogenicity observed during the Brazilian epidemic.

## 1. Introduction

Zika virus (ZIKV) is an emerging arthropod-borne flavivirus transmitted mainly through *Aedes sp.* mosquito bites. In addition, sexual and maternofetal transmissions have also been documented in recent outbreaks [1]. ZIKV was first identified as a filterable transmissible agent from the serum of a febrile sentinel rhesus macaque in the Ziika forest (later renamed Zika) of Uganda in 1947 [2]. The first human cases of ZIKV infection were reported in 1952, and since then it has slowly spread through Southeast Asia with the first Asian lineage isolate, P6-740, identified in Malaysia in 1966 [3,4]. A large outbreak occurred in 2007 on several islands in the State of Yap, Micronesia, in the Western Pacific, followed by epidemics in French Polynesia, Easter Island, the Cook Islands and New Caledonia in 2013–2015 [5,6]. It reached South America in 2014 resulting in a large outbreak across Brazil in 2015 where ZIKV RNA was detected in people with exanthematous illness and arthralgia [7,8].

In the early epidemics, ZIKV infection was considered a mild disease. Symptoms included a rash, conjunctivitis and mild fever while many infected people had no symptoms [9,10]. By December 2015, the Minister of Health in Brazil revealed increased incidence of neurological complications like Guillain-Barré syndrome (GBS), and a large increase in the number of microcephaly cases in babies born from infected mothers, specifically in areas of high endemic ZIKV circulation [11,12,13,14]. A retrospective analysis in the French Polynesia showed that ZIKV-related GBS and microcephaly also occurred, while there were no or few such reports from the epidemic in Asia [15,16,17]. ZIKV increased pathogenicity and rapid ability to spread in tropical areas of the Americas raise questions regarding whether there is a genetic basis for these changes between the early Asian ZIKV strains and the contemporary Brazilian isolates [17,18].

ZIKV is a flavivirus from the *Flaviviridae* family with similar genome organization to other members such as Dengue, West Nile, yellow fever and Japanese encephalitis viruses [3]. The ZIKV genome is a monocistronic 11 kb positive-sense RNA, which is translated into a single polyprotein. The polyprotein is cleaved by host and viral proteases into three structural proteins (C, prM, E) and seven non-structural proteins (NS1, NS2A, NS2B, NS3, NS4A, NS4B and NS5) [19,20]. The virion size is approximately 50 nm, in which the capsid is surrounded by the structural membrane protein prM/M and the viral envelope E [19]. Compared to other flaviviruses the virion is thermostable and has a more compact surface, which may contribute to its stability in body fluids, such as saliva, urine or semen [21]. Dermal fibroblasts, epidermal keratinocytes and dendritic cells are the first cells to be infected by ZIKV after a mosquito bite [22]. ZIKV also infects human microglia, neural progenitors and astrocytes, as well as human fetal endothelial cells through interactions with the Gas6 ligand and its cellular receptor, AXL. Receptor interactions trigger clathrin-mediated endocytosis and ZIKV capsids are released through fusion of the viral envelope with the endosomal membrane [23,24,25]. While the ZIKV replication cycle proceeds in a similar manner to the related flaviviruses, the specific host-virus interactions important for ZIKV infection are not yet clear [7].

Based on phylogenetic analyses, the current circulating ZIKV strains have evolved from the common African ancestor, MR766, representing the African lineage. A breaking point occurred with strain P6-740 from Malaysia that determined the beginning of the Asian lineage and the current ZIKV circulating in the Americas have spread from Asia [26,27]. Due to the changes between the early Asian disease and the Americas’ epidemics, we studied the virus characteristics of one example from each epidemic [26] to determine if they could bring some explanation to the increased pathogenicity and dissemination. In this paper, we compared the cytopathicity of a Canadian-imported Thai strain of ZIKV representing the early Asian lineage to a Brazilian strain isolated from Bahia in 2015. The Brazilian isolate generated higher cytopathicity and intracellular RNA accumulation in the simian Vero and the human HEK 293T cell lines but higher cytopathicity with similar intracellular RNA accumulation in a neuroblastoma cell line. We performed an amino acid (aa) sequence comparison and predicted α-helices, β-strands and coils content between strains. Further examination of the specific aa polymorphisms may provide some insights on the underlying causes of these phenotypic differences observed in cell culture.

## 2. Materials and Methods

### 2.1. Cell Culture

HEK 293T cells [28,29] were maintained in Dulbecco’s modified Eagle’s medium (DMEM) with high glucose (Hyclone, Logan, UT, USA) supplemented with 10% fetal bovine serum (FBS) (Hyclone, Logan, UT, USA), 50 U/mL Penicillin and 50 μg/mL Streptomycin (Thermo Fisher Scientific, Burlington, ON, Canada). African green monkey epithelial Vero cells [30] were maintained in DMEM with high glucose, supplemented with 5% FBS, 1% non-essential amino acids, 1% L-glutamine, 50 U/mL Penicillin and 50 μg/mL Streptomycin (Wisent, St Bruno, QC, Canada). Neuroblastoma SH-SY5Y cells [31] were obtained from Dr. Marc Fabian (LDI and McGill University, Montréal, QC, Canada) and maintained in DMEM/Ham’s F12 50/50 mix (Wisent, St Bruno, QC, Canada), supplemented with 10% FBS, 50 U/mL Penicillin and 50 μg/mL Streptomycin.

### 2.2. Zika Virus Strains

The Canadian imported Thai ZIKV strain PLCal_ZV (Genbank accession KF993678.1), passaged four times in Vero cells was previously described [30,32]. The Brazilian ZIKV strain HS-2015-BA-01 isolated in August 2015 in Salvador, Bahia was previously described (Genbank accession KX520666.1). It was passaged three times in *Aedes albopictus* C6/36 mosquito cells and once in Vero cells [33].

### 2.3. Zika Virus Amplification 

ZIKV stocks were prepared by passaging in Vero cells. Briefly, 6.0 × 10^6^ Vero cells were plated in a T182.5 flask. On day 1, the growth medium was removed and cells were washed with Phosphate Buffer Saline (PBS) (Wisent, St Bruno, QC, Canada). Cells were then infected at a multiplicity of infection (MOI) of 0.5 in 10 mL of Eagle’s minimal essential medium (EMEM) (Wisent, St Bruno, QC, Canada) and incubated (37 °C, 5% CO_2_) for 2 h. The infection medium was then removed and replaced with ZIKV infection medium: DMEM supplemented with 2% FBS; 1% non-essential amino acids; 1% L-glutamine; 50 U/mL Penicillin and 50 μg/mL Streptomycin (Wisent, St Bruno, QC, Canada), and; 15 mM Hepes buffer (Sigma-Aldrich, Oakville, ON, Canada). At two days post-infection, the supernatant was filtered through a 0.45 μM membrane, viral stocks were tittered by plaque forming unit (PFU) assay, aliquoted and stored at −80 °C.

### 2.4. Live Cell Imaging of Zika Virus Infection

For this, 3.0 × 10^5^ Vero, HEK 293T and SH-SY5Y cells were seeded in 12-well plates and incubated overnight. Growth medium was removed and cells were washed with PBS. Cells were infected at MOI of 0.1 in 1 mL of EMEM with either ZIKV PLCal_ZV or ZIKV HS-2015-BA-01 and incubated for 2 h. The infection medium was replaced with supplemented ZIKV infection medium in DMEM or DMEM/Ham’s F12 50/50 mix. The infection was followed for 6, 12, 24 and 48 h to observe and visualize the cytopathic effects. Capture imaging of live cells was acquired with a ZOE cell imager (Bio-Rad, Mississauga, ON, Canada).

### 2.5. Plaque Forming Unit (PFU) Assay 

At this stage, 6.0 × 10^5^ Vero cells were seeded in six-well plates and incubated overnight. On day 1, eight serial dilutions (1 in 10) were performed with the supernatant filtrates in EMEM. The medium was removed, cells were washed with PBS and then incubated with the virus dilutions for 2 h. After incubation, the virus dilutions were removed and replaced with a mixture of: 1.2% carboxymethylcellulose (CMC) (Sigma-Aldrich), 2% FBS and 50 U/mL Penicillin and 50 μg/mL Streptomycin in EMEM. Four days post-infection, the CMC medium was removed, and after two washes with PBS, cells were fixed with 2 mL of a 4% paraformaldehyde (PFA) solution, washed with ddH_2_O and incubated at room temperature (RT) with a 0.1% crystal violet solution for 30 min to visualize plaques. Viral titers were calculated as follows:

$$\frac{\#\;of\;plaques}{dilution\;factor\;\times \;infection\;volume}$$

### 2.6. Quantitative Reverse Transcription Polymerase Chain Reaction (qRT-PCR)

RNA was extracted from Vero, HEK 293T and SH-SY5Y cell lines mock infected or infected with PLCal_ZV or HS-2015-BA-01 at MOI of 0.01, 0.1 and 0.5 for 24 h with Trizol reagent (Life Technologies, Burlington, ON, Canada) and further purified with RNeasy (QIAGEN, Hilden, Germany). RNase-Free DNase Set (QIAGEN) was loaded onto the RNeasy columns. cDNA was synthesized using 1000 ng of the extracted RNA using Superscript II according to the manufacturer’s protocol (Invitrogen, Burlington, ON, Canada). qPCR was performed by diluting the cDNA (1:60) due to the comparison with the threshold cycle (Ct) values from the ZIKV standard curve. The standard curve was generated by serial dilution of pooled samples of infected cell lines with their respected viruses. BrightGreen qPCR MasterMix-Low ROX and a Bio-Rad CFX96 were used for performing the qPCR. Primers for TATA-box binding protein (TBP) and Peptidylprolyl isomerase A (PPIA) were used as internal controls as previously described [34]. ZIKV primers were designed for this work with the following sequences: Forward 5′-CAAAAGGAGGCCCTGGTCAT-3′, Reverse 5′-ATGAAAGACGTCCACCCCAC-3′ (92 bp product). Samples were loaded in quadruplicates. qPCR conditions were as follows: 95 °C for 5 min followed by 50 cycles of 95 °C for 10 s, 62 °C for 15 s, 72 °C for 5 s. Finally, one cycle of 65 °C for 5 s and one of 95 °C for 5 s. Data analysis was performed using Bio-Rad CFX software (http://www.bio-rad.com/en-ca/category/qpcr-analysis-software) and GraphPad Prism 5 (GraphPad Software, La Jolla, CA, USA).

### 2.7. Cell Viability

Cell viability was evaluated by the metabolism of the water-soluble tetrazolium salt 1 (WST-1) cell proliferation reagent (Roche, Indianapolis, IN, USA). Briefly, 2.5 × 10^4^ Vero, HEK 293T, and Neuroblastoma SH-SY5Y cells were seeded in 96-well plates and incubated overnight. On day 1, the growth medium was removed and cells were washed with PBS. Cells were then infected with either PLCal_ZV or HS-2015-BA-01 ZIKV isolates at an MOI of 1, 0.5, 0.1, and 0.01 in 100 μL of EMEM. The cells were incubated with the virus dilutions for 2 h. Virus solutions were then removed and replaced with 100 μL of the respective growth media. 24 and 48 h post-infection, 50 μL of Dimethyl sulfoxide (DMSO) (Sigma-Aldrich, Oakville, ON, Canada) was added to four wells of each cell line as a control for cell death. After 15 min incubation at RT, 10 μL of WST-1 reagent was added to each well and cells were incubated for an additional 1.5 h at 37 °C. ZIKV was then neutralized for 20 min by the addition of 50 μL PBS with 4% NP40 (Sigma-Aldrich, Oakville, ON, Canada). The plate was then read on a Benchmark Plus microplate spectrophotometer (Bio-Rad, Mississauga, ON, Canada). Absorbance was measured at 450 nm (test wavelength) and 690 nm (reference wavelength) and the reference reading was subtracted from the test reading. The value of the uninfected cells was set as 100% viability. The viability of each condition was expressed as a percentage of the uninfected cells for each cell line. Each condition was performed in triplicates, and the percentage viability for each cell line at each MOI was calculated as follows:

$$\frac{OD_{infected}^{450\;\mathrm{nm}}-OD_{infected}^{690\;\mathrm{nm}}}{OD_{mock}^{450\;\mathrm{nm}}-OD_{mock}^{690\;\mathrm{nm}}}\;\times 100$$

### 2.8. ZIKV Polyprotein Sequence Alignment and Secondary Structure Predictions 

ZIKV DNA sequence from PLCal_ZV and HS-2015-BA-01 were aligned against the ancestor African strain MR766 to define the protein boundaries in Clustal W (Kyoto University Bioinformatics Center, Kyoto, Japan) and Molecular Evolutionary Genetics Analysis, MEGA 6 (The Pennsylvania State University, Pennsylvania, USA). This alignment helped us to define the ZIKV polyprotein sequences of PLCal_ZV and HS-2015-BA-01. Once we defined the precise boundaries of each ZIKV protein, we submitted the aa sequence of each mature protein to PSIPRED (http://bioinf.cs.ucl.ac.uk/psipred/) to predict the overall secondary structure (α-helix, β-strand and coil content).

## 3. Results

### 3.1. Protein Comparison between the Canadian-Imported Thai Strain and the Brazilian Isolate from Bahia Identifies Amino Acid Polymorphisms across the ZIKV Polyproteins

**Figure 1:**
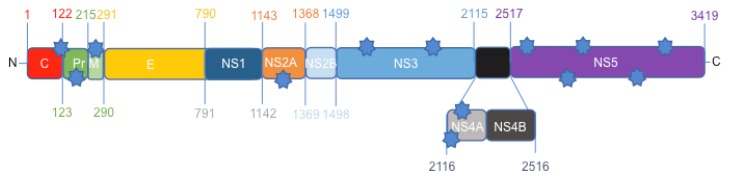
Zika virus (ZIKV) genome representation. Stars through the genome represent the position of aa differences between the Canadian-imported Thai strain PLCal_ZV and the Brazilian strain HS-2015-BA-01.

Based on previous phylogenetic nucleotide analyses, the Canadian-imported Thai strain PLCal_ZV of ZIKV represents an early Asian isolate of the virus before the French Polynesian outbreak [35,36,37,38]. It is far separated from the Brazilian strain HS-2015-BA-01 [26], which is closely related to other isolates from Bahia in the Asian lineage [39]. To investigate the aa difference between these two strains, we first compared the whole sequence of the two polyproteins (Figure S1). This comparison showed that there are only 13 aa polymorphisms between the PLCal_ZV and HS-2015-BA-01 isolates (Figure 1).

The African MR766 reference strain, which is much more divergent, was used for comparison at each of these polymorphisms between PLCal_ZV vs. HS-2015-BA-01 (Table 1). We found 13 aa polymorphisms between the two isolates, and the Brazilian isolate has five of those aa that were identical to the African reference strain. The aa variation between the Thai and Brazilian isolates are in the ER anchor of the C, Pr/PrM, NS2A, NS3, NS4A and NS5 proteins. Interestingly, no difference was found in the viral E protein, which is the main component for viral-cell recognition (Table 1). 

### 3.2. ZIKV Brazilian Isolate Demonstrates Increased Cytopathic Effects When Compared to the Asian Thai Strain in Three Different Cell Lines

**Figure 2:**
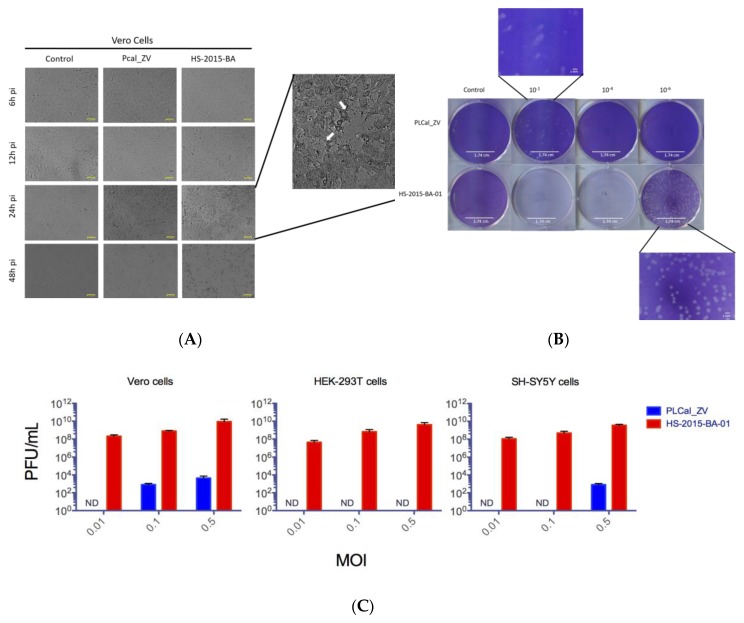
ZIKV HS-2015-BA is more cytopathic than PLCal_ZV in Vero, HEK 293T and SH-SY5Y cells. (**A**) Kinetics of cytopathicity of ZIKV in Vero cells. Vero cells were infected at multiplicity of infection (MOI) 0.1 with mock, PLCal_ZV or HS-2015-BA-01 as indicated. Live cells were photographed under the microscope after 6, 12, 24 and 48 h post-infection, scale bar = 63 μm. White arrows show the cytopathic effect of HS-2015-BA on Vero cells; (**B**) plaque assay in Vero cells of ZIKV PLCal_ZV and HS-2015-BA-01 after infection with 10^−1^, 10^−4^ and 10^−6^ dilution of virus from Vero cell supernatants, scale bar = 1.74 cm in the plates and 1 mm in the zoomed pictures; (**C**) quantification of ZIKV plaque forming units (PFUs) formed by PLCal_ZV and HS-2015-BA-01 produced in Vero, HEK 293T and SH-SY5Y cell lines. Supernatant from each infected cell line at MOI of 0.01, 0.1 or 0.5 was collected and filtered on a 0.45 μm pore membrane. The generated supernatant was used to infect new batches of Vero cells and calculate the PFUs for every cell line. Detection limit is 400 PFUs/mL. ND = not detected. The graphs represent the average of three independent experiments ± standard error of the mean (SEM).

ZIKV has a tropism for different cells and has neuronal cytopathic effects [24,33,40,41]. To compare the cytopathicity and the viral titer between the Thai and Brazilian isolates we evaluated their capacity to induce cell death and to generate new viruses in three different cell lines, Vero, HEK 293T and neuroblastoma SH-SY5Y cells. We first performed a time point infection in each cell line and visualized the cells for 6, 12, 24 and 48 h post-infection (Figure 2A). We observed that the cytopathic effects were highly visible starting at 24 h in Vero cells infected by the HS-2015-BA-01 with the majority of Vero cells lysed at 48 h post-infection. In contrast, Vero cell lysis was not observed with the PLCal_ZV strain under these conditions (Figure 2A). We did not observe any modification of HEK 293T or SH-SY5Y cell morphology before 48 h of infection with HS-2015-BA-01. At that time there was cell detachment with partial cell death of HEK 293T and complete cell death of SH-SY5Y. RNA extraction and quantification could not be performed at 48 h due to cell death and the optimal time for further assays was set-up at 24 h.

To amplify and quantify cell lysis, the supernatant of infected cells was collected at 24 h post-infection and used for plaque assays in Vero cells. An example of this assay from the supernatant of Vero cells is shown in Figure 2B. From the virus collected at 24 h, we observed that PLCal_ZV gave plaques at 10^−1^ dilution of virus and no plaques were observed at higher dilution. With HS-2015-BA-01, cells were all lysed with the higher concentrations and plaques were countable only after a 10^−6^ dilution. The shape of the plaques was also different. In the infected Vero cells with PLCal_ZV, the plaques had reproducibly more fuzzy, indefinite boundaries and mostly as a shape of a comet. In contrast, the plaques with HS-2015-BA-01 were consistently round with sharp distinct boundaries, as circles, (Figure 2B, zoomed fields).

Because the two ZIKV strains showed different plaque production at a similar MOI, we quantified the viral cytopathicity generated by both viruses in each cell line at different MOIs (Figure 2C). We observed large differences in viral PFUs generated between PLCal_ZV and HS-2015-BA-01 in all three cell lines with the Brazilian isolate that consistently generated plaque numbers several logs higher at all MOIs. Overall the results show that HS-2015-BA-01 induces higher cytopathic effects than PLCal_ZV in three different cell lines. To determine if the extent of cell killing correlates with RNA accumulation, we next used qRT-PCR to quantify the RNA from the cells infected by each ZIKV strain.

### 3.3. ZIKV Brazilian Isolate Infection Results in Higher Viral RNA Accumulation Compared to Infection with the Thai Strain in Two Cell Lines

**Figure 3:**
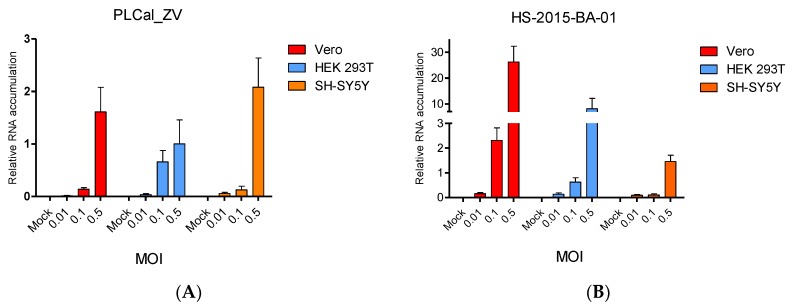
ZIKV intracellular RNA accumulation. Vero (red), HEK 293T (blue) and SH-SY5Y (orange) cells were mock infected or infected with PLCal_ZV (**A**) or HS-2015-BA-01 (**B**) at MOI 0.01, 0.1 or 0.5 as indicated. RNA accumulation was quantified by quantitative reverse transcription polymerase chain reaction (qRT-PCR) at 24 h post-infection. The amount of ZIKV RNA was analyzed by the threshold cycle (Ct) comparative method and normalized to the reference genes TATA-box binding protein (TBP) and Peptidylprolyl isomerase A (PPIA) RNAs using Bio-Rad CFX software. The graphs represent an average of three independent experiments ± standard error of the mean (SEM). GraphPad Prism was used to calculate P-values for effects on RNA accumulation between PLCal_ZV and HS-2015-BA-01 in the different cell lines. Results from a two-way ANOVA were: *p* < 0.0001 for Vero cells, *p* < 0.05 for HEK293T cells and not significant for SH-SY5Y cells.

To further evaluate phenotypic differences between the two ZIKV isolates, we quantified the intracellular viral RNA levels in each of the three cell lines used in Figure 2. We designed primers to target the NS5 domain in ZIKV (nucleotides 7871–7962) to amplify similarly PLCal_ZV and HS-2015-BA-01 viral RNAs by qRT-PCR assay and quantified viral RNA accumulation at 24 h post-infection (Figure 3).

Our results show that in Vero and HEK 293T cell lines infected with HS-2015-BA-01, the amount of viral genomic RNA was higher than with PLCal_ZV at all MOIs. Interestingly, the RNA level at MOI 0.5 was 16 times higher in Vero cells and 10 times higher in HEK 293T cells with HS-2015-BA-01 than with PLCal_ZV. Despite the increased cytopathicity of HS-2015-BA-01 in the SH-SY5Y cell line (Figure 2), the RNA accumulation from the two viral strains was similar at 24 h post-infection. Overall, the level of viral RNA is higher with HS-2015-BA-01 than with PLCal_ZV at 24 h in Vero and HEK 293T cells, but no apparent difference was observed by qRT-PCR in SH-SY5Y. Therefore, the cytopathicity may involve another parameter different from RNA accumulation, at least in the neuroblastoma SH-SY5Y cells.

### 3.4. ZIKV Brazilian Isolate Decreases Cell Viability More than the Thai Strain in All Three Cell Lines

**Figure 4:**
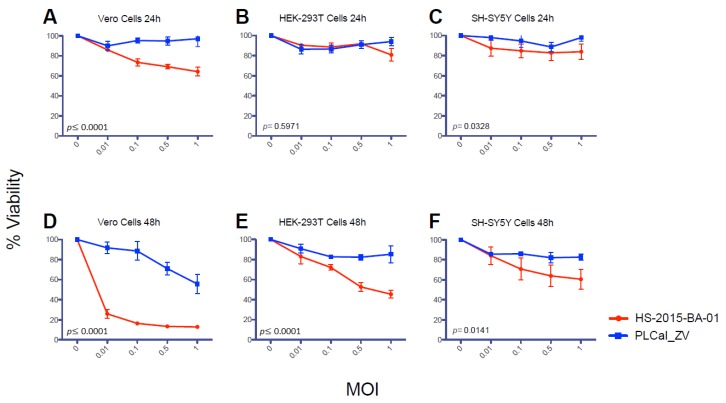
Cellular viability assays in different cells infected with PLCal_ZV or HS-2015-BA-01. Cells were infected with PLCal_ZV (blue) or HS-2015-BA-01 (red). (**A**–**C**) Cell viability was measured at 24 h in Vero (**A**); HEK 293T (**B**) and SH-SY5Y (**C**); (**D**–**F**) Cell viability assays at 48 h in Vero (**D**); HEK 293T (**E**) and SH-SY5Y (**F**). The OD 450 nm of the mock was set as 100% viability, and each condition was expressed as a percentage of the mock. The values are the average of three independent experiments ± standard error of the mean (SEM). GraphPad Prism was used to calculate *p*-values for effects on cell viability between PLCal_ZV and HS-2015-BA-01. Results from a two-way ANOVA are shown on each graph, **A**–**F**.

A different way to assess cytopathicity of the ZIKV strains is to measure their effect on the cellular metabolism. We used a cell viability assay based on the cleavage of the tetrazolium salt, WST-1, into a formazan dye. This reaction is dependent on NAD(P)H and is only intact in metabolically active cells. Vero, HEK 293T and SH-SY5Y cells were infected with PLCal_ZV and HS-2015-BA-01 viruses at increasing MOI. We observed that the HS-2015-BA-01 decreases cell viability of Vero cells more than the PLCal_ZV at 24 h, while the differences between the two strains were negligible for the HEK 293T and SH-SY5Y cells (Figure 4A–C). At 48 h, we see a large difference between the two strains in all three cell lines tested, most notably in the Vero cells (Figure 4D–F). This result shows an increased effect of HS-2015-BA-01 on reducing cell viability compared to PLCal_ZV and is consistent with results from plaque assays (Figure 2C) and from qRT-PCR (Figure 3).

### 3.5. ZIKV Secondary Structure of prM, NS2A, NS3, and NS5 Produce Different Patterns of Predicted α-Helices, β-Strands and Coils

To determine if the aa differences between the two ZIKV strains could change the structure of the corresponding proteins, we analyzed the predicted protein secondary structure using a protein prediction program (http://bioinf.cs.ucl.ac.uk/psipred/). The structures were analyzed by comparing the α-helices, β-strands and coils between proteins of PLCal_ZV and HS-2015-BA-01 (Table 2 and Figure S2).

The aa modifications in the ER anchor of the Core protein (T106A) and in the NS4A protein (F2123L and L2167M) induced no change of their predicted secondary structures and have little chances to change their properties. The two aa changes in prM (S139N and S273R) and one in NS2A (A1263V) induced several predicted modifications in the secondary structures with several inversions between β-strands and coils (prM) or α-helices and coils (NS2A). Furthermore, the two aa changes in NS3 and five in NS5 induced many differences between predicted coils, α-helices or β-strands. Interestingly, in several cases the aa variation induced an upstream or downstream modification of the predicted secondary structure suggesting that the substructures are interconnected and could affect overall protein folding.

## 4. Discussion

The rapid dissemination of ZIKV and increased pathogenicity between the early Asian strains and the contemporary American isolates prompted us to determine if a difference in cytopathicity and viral RNA accumulation could provide some insight into novel neurological pathogenesis observed [11,12,13,14,17,18]. We therefore chose to study the Canadian-imported Thai strain PLCal_ZV, which belongs to the early Asian lineage and HS-2015-BA-01, a recent Brazilian isolate from Bahia, a region where fetal pathogenesis was detected early in the epidemic [18,32,33,39,42]. An aa comparison between the two strains identified 13 aa polymorphism that may contribute to strain phenotypic variations (Figure 1).

ZIKV has a tropism for many human cells and tissues [43]. It can infect the skin fibroblasts, keratinocytes and dendritic cells [22], monocytes [44,45], retinal epithelium [46], brain cells including neural progenitors, neurons, astrocytes and glial cells [23,41,47,48], uterine fibroblasts [49], placental cells [50] and testicular cells [51,52]. In cell culture, insect C6/36 and monkey epithelial Vero cell lines have been widely used to amplify ZIKV [30,53], whereas human fibroblasts MRC-5, hepatic Huh7, lung epithelial cells A549, astrocytic U251MG, neuronal stem cells and neuroblastoma SK-N-SH and SH-SY5Y cell lines are permissive for ZIKV infection [40,53,54,55,56]. Human epithelial HEK 293T cells were found to be moderately permissive to ZIKV infection [22,40]. To better understand the cytopathicity of the two different ZIKV strains, we infected the monkey epithelial Vero and the human epithelial HEK 293T cell lines. Because the main concern about ZIKV is its neurological consequences in fetuses and adults, we used the human SH-SY5Y neuroblastoma cell line as a model for neuronal infection and cytopathicity [16,48,57]. These cellular models showed greater cytopathic effects with HS-2015-BA-01 than with PLCal_ZV as noticed by live cell observation at different time points and quantification of cell lysis (Figure 2). Furthermore, measurement of cell viability demonstrated that HS-2015-BA-01 induced more metabolic damage than PLCal_ZV in all three cell lines (Figure 4). One explanation could be a different tropism related to the entry receptors. AXL is the main ZIKV candidate receptor, present in several cell types such as keratinocytes, HEK 293T, neuronal stem cells, astrocytes, oligodendrocyte precursor cells, microglia and endothelial cells, but its removal does not protect from ZIKV infection suggesting that others are involved [22,23,25,47,58,59,60]. Indeed, Tyro3 and T-cell immunoglobulin and mucin domain (TIM)1 could be part of the ZIKV entry receptors or co-receptors that enhance the virus entry either alone or with AXL [43,50]. However, the fact that the E protein has no aa polymorphism does not favor this hypothesis. The intracellular milieu may also influence the cytopathic effects of the virus by interactions with cellular components. Those include helper or restriction factors, cellular trafficking, RNA replication and translation of viral proteins that either favor or restrict ZIKV replication [40,61,62,63,64]. The immune response capabilities of each cell line studied can also influence the replication rate or cytopathic effects [30,65]. Specific viral proteins can also interact with cellular components and induce metabolic disturbances in cells, which could lead to pathogenesis, decreased cell growth and ultimately cell death [33,48,54,56,66].

In addition, the difference in cytopathicity could have its origin in the extent of viral replication, which is the result of both cellular and viral factors. To approach viral replication after viral entry, we measured the intracellular accumulation of viral RNA. Our results show that genomic RNA levels of HS-2015-BA-01 are greater than that of PLCal_ZV in Vero and in HEK 293T cells at 24 h (Figure 3). In neuroblastoma SH-SY5Y cells, at each MOI tested, the viral RNA levels were similar between the two strains, indicating that the amount of viral RNA accumulation cannot explain the increased cytopathicity of the Brazilian isolate in these cells. Previous studies that compared strains from the African and the Asian lineage have reported a higher replication rate and cytopathicity for the African lineage in different cell lines [67]. Specifically, in a neuroblastoma cell line SK-N-SH (the precursor of SH-SY5Y), two African strains replicated faster than two Asian strains, but the viral RNA produced was not quantified [35]. When qRT-PCR was performed to compare the American isolate from Puerto Rico to the African MR766 strain, a higher RNA production was found for the Puerto Rico strain in two endothelial cells but no neuronal cells were used in this study [58]. Therefore, this is the first report that shows higher viral RNA accumulation and higher cytopathicity for a Brazilian isolate compared to an early Asian Thai strain in the endothelial monkey (Vero) and human (HEK 293T) cells, whereas in the neuroblastoma cells SH-SY5Y the cytopathicity of the Brazilian isolate is higher with similar intracellular RNA levels (Figure 2, Figure 3 and Figure 4). These results suggest that some neuropathology may not be linked to higher viral RNA accumulation but rather to a specific activity of one or several viral proteins.

Previous studies have shown that aa polymorphism in the ZIKV polyprotein can alter cell tropism, virulence or the replication rate of viruses. For example, a substitution of five aa in ZIKV has contributed to the adaptation from mosquitoes to human and a single aa substitution in NS1 was associated with increased infectivity in mosquitoes [68,69,70]. Here we found no further changes in the E and NS1 proteins between PLCal_ZV and HS-2015-BA-01 strains, suggesting that the increased cytopathicity and differences in viral RNA levels may be due to polymorphisms in other ZIKV proteins (Figure 1 and Table 1). We therefore compared the predicted secondary structure of each modified protein (Figure S2 summarized in Table 2). In the PrM protein, S139N and S273R induced modifications that are predicted to cause several changes between β-strands and coil structures. A recent report comparing Americas’ isolates to an early Asian strain from Cambodia identified S139N as a major mutation that contributes to fetal microcephaly in a mouse model [66]. The prM protein is involved in virus maturation and secretion. The higher viral production despite similar RNA levels in SH-SY5Y for the HS-2015-BA-01 compared to PLCal_ZV strain suggests that S139N polymorphism in prM may contribute to increased viral egress and dissemination and consequently to cytopathicity and neuropathology. Additional polymorphisms of the ZIKV proteins were identified in NS2A, NS3 and NS5. The Y2086H polymorphism in NS3 protease and helicase occurred between ZIKV PLCal_ZV and THA/2014/SV0127-14, another Thai isolate [20,32,37,71]. It remains to be determined if this mutation has changed the proteolytic activity of NS3 in the subsequent isolates. NS5 acts as a methyl transferase in its N-terminus and as an RNA-dependent RNA polymerase in its C-terminus [72]. Mutation M2634V in NS5 was identified in all Latin American viruses but not in French Polynesia isolates. Further studies will be required to determine if it had an incidence on NS5 activity in the Americas’ isolates [37]. Although the 5′ and 3′ RNA elements could also play critical roles in the viral replication cycle [20], our study focused on the aa sequence changes and their impacts on predicted secondary structures. Their consequences on increased cytopathicity may contribute to neuropathology. HS-2015-BA-01 has been used in vivo in a murine model and in a primate model [33,65]. The virus kills fetal mice neurons very efficiently [33], but adult monkeys show an efficient immune reaction against the virus with no major pathology [65]. Further research will address the main determinants of placenta transmission, teratogenesis and neuropathology.

## Figures and Tables

**Table 1:** Amino acid comparison of ZIKV MR766 at the point mutations between PLCal_ZV and HS-2015-BA-01.

Strain	Amino acid position												
MR-766	A106	A139	S273	A1263	D1622	Y2086	L2123	L2167	Y2594	M2634	V2842	V2894	P3162
PLCal_ZV	T106	S139	S273	A1263	D1622	Y2086	F2123	L2167	Y2594	M2634	I2842	I2894	S3162
HS-2015-BA-01	A106	N139	R273	V1263	G1622	H2086	L2123	M2167	H2594	V2634	V2842	V2894	P3162
Protein	ER anchor	pr/prM	prM	NS2A	NS3	NS3	NS4A	NS4A	NS5	NS5	NS5	NS5	NS5

Red: amino acid (aa) that differ from MR766. Blue: ZIKV proteins.

**Table 2:** Changes in predicted protein structure between PLCal_ZV and HS-2015-BA-01 ZIKV.

Observed changes in PLCal_ZV and HS-2015-BA-01			
*Protein*	*AA Variant position*	*PL Cal_ZV structure*	*HS-2015-BA-01 structure*
pr/prM	139	Coil (171–174, 187, 202, 203)	β-strand (171–174, 187, 202, 203)
	273	β-strand (235, 236)	Coil (235, 236)
NSA2	1263	α-helix (1172, 1214)	Coil (1172, 1214)
		Coil (1232)	α-helix (1232)
NS3	1622	β-strand (1508, 1520, 1524, 1565, 1614, 1641, 1642, 1788, 1947–1949, 2095)	Coil (1508, 1520, 1524, 1565, 1614, 1641, 1642, 1788, 1947–1949, 2095)
	2086	α-helix (1773, 1854, 1855, 1994–1997)	Coil (1773, 1854, 1855, 1994–1997)
		Coil (1625, 2066)	β-strand (1625, 2066)
		Coil (1794, 1958)	α-helix (1794, 1958)
		α-helix (1998, 1999)	β-strand (1998, 1999)
NS5	2594	β-strand (2554, 2555, 2600, 2970, 2997, 3237, 3240, 3241)	Coil (2554, 2555, 2600,2970, 2997, 3237, 3240, 3241)
	2634	Coil (2607, 2608, 3270, 3271, 3287)	α-helix (2607, 2608, 3270, 3271, 3287)
	2842, 2894	Coil (3110)	β-strand (3110)
	3162	α-helix (3159, 3160)	Coil (3159, 3160)

## Supplementary Materials

The following are available online at http://www.mdpi.com/1999-4915/10/2/53/s1, Figure S1: Amino acids sequence comparison between the ZIKV Canadian-imported Thai strain PLCal_ZV and the Brazilian HS-2015-BA-01 strain. Figure S2: Protein secondary structure comparison between the ZIKV Canadian-imported Thai strain PLCal_ZV and the Brazilian HS-2015-BA-01 strain.
