# Supplementary material for: Higher Cytopathic Effects of a Zika Virus Brazilian Isolate from Bahia Compared to a Canadian-Imported Thai Strain
Source: Viruses. 2018 Jan 27;10(2):53. doi: 10.3390/v10020053 (PMC5850360; doi:10.3390/v10020053)
Supplement: Supplementary file 1 [file viruses-10-00053-s001.pdf]

Higher virus production and cytotoxic effects of a Zika virus Brazilian isolate from Bahia compared to a Canadian-imported Thai strain

Sergio P. Alpuche-Lazcano, Craig R. McCullogh, Olivier Del Corpo, Elodie Rance, Robert J. Scarborough, Andrew J. Mouland, Selena M. Sagan, Mauro M. Teixeira and Anne Gatignol

Supplementary Materials:

Figure S1: Amino acids sequence comparison between the ZIKV Canadian-imported Thai strain PLCal_ZV and the Brazilian HS-2015-BA-01 strain. The 5' and 3' ends of the nucleotide sequence of PLCal_ZV reported in GenBank are not completely sequenced; therefore the 37aa at the N-terminus and the 6 aa at the C-terminus of the polyproteins could not be aligned.

```
PLCal_ZV           -------------------------------------LLGHGPIRMVLAILAFLRFTAIK
HS-2015-BA-01      MKNPKKKSGGFRIVNMLKRGVARVSPFGGLKRLPAGLLLGHGPIRMVLAILAFLRFTAIK
                                                        ***********************

PLCal_ZV           PSLGLINRWGSVGKKEAMEIIKKFKKDLAAMLRIINARKEKKRRGTDTSVGIVGLLLTTA
HS-2015-BA-01      PSLGLINRWGSVGKKEAMEIIKKFKKDLAAMLRIINARKEKKRRGADTSVGIVGLLLTTA
                   *********************************************:**************

PLCal_ZV           MAAEVTRRGSAYYMYLDRSDAGEAISFPTTLGMNKCYIQIMDLGHMCDATMSYECPMLDE
HS-2015-BA-01      MAAEVTRRGSAYYMYLDRNDAGEAISFPTTLGMNKCYIQIMDLGHMCDATMSYECPMLDE
                   ******************.*****************************************

PLCal_ZV           GVEPDDVDCWCNTTSTWVVYGTCHHKKGEARRSRRAVTLPSHSTRKLQTRSQTWLESREY
HS-2015-BA-01      GVEPDDVDCWCNTTSTWVVYGTCHHKKGEARRSRRAVTLPSHSTRKLQTRSQTWLESREY
                   ************************************************************

PLCal_ZV           TKHLIRVENWIFRNPGFALAAAAIAWLLGSSTSQKVIYLVMILLIAPAYSIRCIGVSNRD
HS-2015-BA-01      TKHLIRVENWIFRNPGFALAAAAIAWLLGSSTRQKVIYLVMILLIAPAYSIRCIGVSNRD
                   ******************************** ***************************

PLCal_ZV           FVEGMSGGTWVDVVLEHGGCVTVMAQDKPTVDIELVTTTVSNMAEVRSYCYEASISDMAS
HS-2015-BA-01      FVEGMSGGTWVDVVLEHGGCVTVMAQDKPTVDIELVTTTVSNMAEVRSYCYEASISDMAS
                   ************************************************************

PLCal_ZV           DSRCPTQGEAYLDKQSDTQYVCKRTLVDRGWGNGCGLFGKGSLVTCAKFACSKKMTGKSI
HS-2015-BA-01      DSRCPTQGEAYLDKQSDTQYVCKRTLVDRGWGNGCGLFGKGSLVTCAKFACSKKMTGKSI
                   ************************************************************

PLCal_ZV           QPENLEYRIMLSVHGSQHSGMIVNDTGHETDENRAKVEITPNSPRAEATLGGFGSLGLDC
HS-2015-BA-01      QPENLEYRIMLSVHGSQHSGMIVNDTGHETDENRAKVEITPNSPRAEATLGGFGSLGLDC
                   ************************************************************

PLCal_ZV           EPRTGLDFSDLYYLTMNNKHWLVHKEWFHDIPLPWHAGADTGTPHWNNKEALVEFKDAHA
HS-2015-BA-01      EPRTGLDFSDLYYLTMNNKHWLVHKEWFHDIPLPWHAGADTGTPHWNNKEALVEFKDAHA
                   ************************************************************

PLCal_ZV           KRQTVVVLGSQEGAVHTALAGALEAEMDGAKGRLSSGHLKCRLKMDKLRLKGVSYSLCTA
HS-2015-BA-01      KRQTVVVLGSQEGAVHTALAGALEAEMDGAKGRLSSGHLKCRLKMDKLRLKGVSYSLCTA
                   ************************************************************
```

```
PLCal_ZV        AFTFTKIPAETLHGTVTVEVQYAGTDGPCKVPAQMAVDMQTLTPVGRLITANPVITESTE
HS-2015-BA-01   AFTFTKIPAETLHGTVTVEVQYAGTDGPCKVPAQMAVDMQTLTPVGRLITANPVITESTE
                ************************************************************

PLCal_ZV        NSKMMLELDPPFGDSYIVIGVGEKKITHHWHRSGSTIGKAFEATVRGAKRMAVLGDTAWD
HS-2015-BA-01   NSKMMLELDPPFGDSYIVIGVGEKKITHHWHRSGSTIGKAFEATVRGAKRMAVLGDTAWD
                ************************************************************

PLCal_ZV        FGSVGGALNSLGKGIHQIFGAAFKSLFGGMSWFSQILIGTLLMWLGLNTKNGSISLMCLA
HS-2015-BA-01   FGSVGGALNSLGKGIHQIFGAAFKSLFGGMSWFSQILIGTLLMWLGLNTKNGSISLMCLA
                ************************************************************

PLCal_ZV        LGGVLIFLSTAVSADVGCSVDFSKKETRCGTGVFVYNDVEAWRDRYKYHPDSPRRLAAAV
HS-2015-BA-01   LGGVLIFLSTAVSADVGCSVDFSKKETRCGTGVFVYNDVEAWRDRYKYHPDSPRRLAAAV
                ************************************************************

PLCal_ZV        KQAWEDGICGISSVSRMENIMWRSVEGELNAILEENGVQLTVVVGSVKNPMWRGPQRLPV
HS-2015-BA-01   KQAWEDGICGISSVSRMENIMWRSVEGELNAILEENGVQLTVVVGSVKNPMWRGPQRLPV
                ************************************************************

PLCal_ZV        PVNELPHGWKAWGKSYFVRAAKTNNSFVVDGDTLKECPLKHRAWNSFLVEDHGFGVFHTS
HS-2015-BA-01   PVNELPHGWKAWGKSYFVRAAKTNNSFVVDGDTLKECPLKHRAWNSFLVEDHGFGVFHTS
                ************************************************************

PLCal_ZV        VWLKVREDYSLECDPAVIGTAVKGKEAVHSDLGYWIESEKNDTWRLKRAHLIEMKTCEWP
HS-2015-BA-01   VWLKVREDYSLECDPAVIGTAVKGKEAVHSDLGYWIESEKNDTWRLKRAHLIEMKTCEWP
                ************************************************************

PLCal_ZV        KSHTLWTDGIEESDLIIPKSLAGPLSHHNTREGYRTQMKGPWHSEELEIRFEECPGTKVH
HS-2015-BA-01   KSHTLWTDGIEESDLIIPKSLAGPLSHHNTREGYRTQMKGPWHSEELEIRFEECPGTKVH
                ************************************************************

PLCal_ZV        VEETCGTRGPSLRSTTASGRVIEEWCCRECTMPPLSFRAKDGCWYGMEIRPRKEPESNLV
HS-2015-BA-01   VEETCGTRGPSLRSTTASGRVIEEWCCRECTMPPLSFRAKDGCWYGMEIRPRKEPESNLV
                ************************************************************

PLCal_ZV        RSMVTAGSTDHMDHFSLGVLVILLMVQEGLKKRMTTKIIISTSMAVLVAMILGGFSMSDL
HS-2015-BA-01   RSMVTAGSTDHMDHFSLGVLVILLMVQEGLKKRMTTKIIISTSMAVLVAMILGGFSMSDL
                ************************************************************

PLCal_ZV        AKLAILMGATFAEMNTGGDVAHLALIAAFKVRPALLVSFIFRANWTPRESMLLALASCLL
HS-2015-BA-01   AKLAILMGATFAEMNTGGDVAHLALIAAFKVRPALLVSFIFRANWTPRESMLLALASCLL
                ************************************************************

PLCal_ZV        QTAISALEGDLMVLINGFALAWLAIRAMVVPRTDNITLAILAALTPLARGTLLVAWRAGL
HS-2015-BA-01   QTVISALEGDLMVLINGFALAWLAIRAMVVPRTDNITLAILAALTPLARGTLLVAWRAGL
                **.*********************************************************

PLCal_ZV        ATCGGFMLLSLKGKGSVKKNLPFVMALGLTAVRLVDPINVVGLLLLTRSGKRSWPPSEVL
HS-2015-BA-01   ATCGGFMLLSLKGKGSVKKNLPFVMALGLTAVRLVDPINVVGLLLLTRSGKRSWPPSEVL
                ************************************************************

PLCal_ZV        TAVGLICALAGGFAKADIEMAGPMAAVGLLIVSYVVSGKSVDMYIERAGDITWEKDAEVT
HS-2015-BA-01   TAVGLICALAGGFAKADIEMAGPMAAVGLLIVSYVVSGKSVDMYIERAGDITWEKDAEVT
                ************************************************************

PLCal_ZV        GNSPRLDVALDESGDFSLVEDDGPPMREIILKVVLMTICGMNPIAIPFAAGAWYVYVKTG
HS-2015-BA-01   GNSPRLDVALDESGDFSLVEDDGPPMREIILKVVLMTICGMNPIAIPFAAGAWYVYVKTG
                ************************************************************

PLCal_ZV        KRSGALWDVPAPKEVKKGETTDGVYRVMTRRLLGSTQVGVGVMQEGVFHTMWHVTKGSAL
HS-2015-BA-01   KRSGALWDVPAPKEVKKGETTDGVYRVMTRRLLGSTQVGVGVMQEGVFHTMWHVTKGSAL
                ************************************************************
```

```
PLCal_ZV          RSGEGRLDPYWGDVKQDLVSYCGPWKLDAAWDGHSEVQLLAVPPGERARNIQTLPGIFKT
HS-2015-BA-01     RSGEGRLDPYWGDVKQDLVSYCGPWKLDAAWDGHSEVQLLAVPPGERARNIQTLPGIFKT
                  ************************************************************

PLCal_ZV          KDGDIGAVALDYPAGTSGSPILDKCGRVIGLYGNGVVIKNGSYVSAITQGRREEETPVEC
HS-2015-BA-01     KGGDIGAVALDYPAGTSGSPILDKCGRVIGLYGNGVVIKNGSYVSAITQGRREEETPVEC
                  *.**********************************************************

PLCal_ZV          FEPSMLKKKQLTVLDLHPGAGKTRRVLPEIVREAIKTRLRTVILAPTRVVAAEMEEALRG
HS-2015-BA-01     FEPSMLKKKQLTVLDLHPGAGKTRRVLPEIVREAIKTRLRTVILAPTRVVAAEMEEALRG
                  ************************************************************

PLCal_ZV          LPVRYMTTAVNVTHSGTEIVDLMCHATFTSRLLQPIRVPNYNLYIMDEAHFTDPSSIAAR
HS-2015-BA-01     LPVRYMTTAVNVTHSGTEIVDLMCHATFTSRLLQPIRVPNYNLYIMDEAHFTDPSSIAAR
                  ************************************************************

PLCal_ZV          GYISTRVEMGEAAAIFMTATPPGTRDAFPDSNSPIMDTEVEVPERAWSSGFDWVTDHSGK
HS-2015-BA-01     GYISTRVEMGEAAAIFMTATPPGTRDAFPDSNSPIMDTEVEVPERAWSSGFDWVTDHSGK
                  ************************************************************

PLCal_ZV          TVWFVPSVRNGNEIAACLTKAGKRVIQLSRKTFETEFQKTKHQEWDFVVTTDISEMGANF
HS-2015-BA-01     TVWFVPSVRNGNEIAACLTKAGKRVIQLSRKTFETEFQKTKHQEWDFVVTTDISEMGANF
                  ************************************************************

PLCal_ZV          KADRVIDSRRCLKPVILDGERVILAGPMPVTHASAAQRRGRIGRNPNKPGDEYLYGGGCA
HS-2015-BA-01     KADRVIDSRRCLKPVILDGERVILAGPMPVTHASAAQRRGRIGRNPNKPGDEYLYGGGCA
                  ************************************************************

PLCal_ZV          ETDEDHAHWLEARMLLDNIYLQDGLIASLYRPEADKVAAIEGEFKLRTEQRKTFVELMKR
HS-2015-BA-01     ETDEDHAHWLEARMLLDNIYLQDGLIASLYRPEADKVAAIEGEFKLRTEQRKTFVELMKR
                  ************************************************************

PLCal_ZV          GDLPVWLAYQVASAGITYTDRRWCFDGTTNNTIMEDSVPAEVWTRYGEKRVLKPRWMDAR
HS-2015-BA-01     GDLPVWLAYQVASAGITYTDRRWCFDGTTNNTIMEDSVPAEVWTRHGEKRVLKPRWMDAR
                  *********************************************:**************

PLCal_ZV          VCSDHAALKSFKEFAAGKRGAAFGVMEALGTLPGHMTERFQEAIDNLAVLMRAETGSRPY
HS-2015-BA-01     VCSDHAALKSFKEFAAGKRGAALGVMEALGTLPGHMTERFQEAIDNLAVLMRAETGSRPY
                  **********************:*************************************

PLCal_ZV          KAAAAQLPETLETIMLLGLLGTVSLGIFFVLMRNKGIGKMGFGMVTLGASAWLMWLSEIE
HS-2015-BA-01     KAAAAQMPETLETIMLLGLLGTVSLGIFFVLMRNKGIGKMGFGMVTLGASAWLMWLSEIE
                  ******:*****************************************************

PLCal_ZV          PARIACVLIVVFLLLVVLIPEPEKQRSPQDNQMAIIIMVAVGLLGLITANELGWLERTKS
HS-2015-BA-01     PARIACVLIVVFLLLVVLIPEPEKQRSPQDNQMAIIIMVAVGLLGLITANELGWLERTKS
                  ************************************************************

PLCal_ZV          DLSHLMGRREEGATIGFSMDIDLRPASAWAIYAALTTFITPAVQHAVTTSYNNYSLMAMA
HS-2015-BA-01     DLSHLMGRREEGATIGFSMDIDLRPASAWAIYAALTTFITPAVQHAVTTSYNNYSLMAMA
                  ************************************************************

PLCal_ZV          TQAGVLFGMGKGMPFYAWDFGVPLLMIGCYSQLTPLTLIVAIILLVAHYMYLIPGLQAAA
HS-2015-BA-01     TQAGVLFGMGKGMPFYAWDFGVPLLMIGCYSQLTPLTLIVAIILLVAHYMYLIPGLQAAA
                  ************************************************************

PLCal_ZV          ARAAQKRTAAGIMKNPVVDGIVVTDIDTMTIDPQVEKKMGQVLLIAVAVSSAILSRTAWG
HS-2015-BA-01     ARAAQKRTAAGIMKNPVVDGIVVTDIDTMTIDPQVEKKMGQVLLIAVAVSSAILSRTAWG
                  ************************************************************

PLCal_ZV          WGEAGALITAATSTLWEGSPNKYWNSSTATSLCNIFRGSYLAGASLIYTVTRNAGLVKRR
HS-2015-BA-01     WGEAGALITAATSTLWEGSPNKYWNSSTATSLCNIFRGSYLAGASLIYTVTRNAGLVKRR
                  ************************************************************
```

```
PLCal_ZV        GGGTGETLGEKWKARLNQMSALEFYSYKKSGITEVCREEARRALKDGVATGGHAVSRGSA
HS-2015-BA-01   GGGTGETLGEKWKARLNQMSALEFYSYKKSGITEVCREEARRALKDGVATGGHAVSRGSA
                ************************************************************

PLCal_ZV        KLRWLVERGYLQPYGKVIDLGCGRGGWSYYAATIRKVQEVKGYTKGGPGHEEPMLVQSYG
HS-2015-BA-01   KLRWLVERGYLQPHGKVIDLGCGRGGWSYYAATIRKVQEVKGYTKGGPGHEEPVLVQSYG
                *************:***************************************:******

PLCal_ZV        WNIVRLKSGVDVFHMAAEPCDTLLCDIGESSSSPEVEEARTLRVLSMVGDWLEKRPGAFC
HS-2015-BA-01   WNIVRLKSGVDVFHMAAEPCDTLLCDIGESSSSPEVEEARTLRVLSMVGDWLEKRPGAFC
                ************************************************************

PLCal_ZV        IKVLCPYTSTMMETLERLQRRYGGGLVRVPLSRNSTHEMYWVSGAKSNTIKSVSTTSQLL
HS-2015-BA-01   IKVLCPYTSTMMETLERLQRRYGGGLVRVPLSRNSTHEMYWVSGAKSNTIKSVSTTSQLL
                ************************************************************

PLCal_ZV        LGRMDGPRRPVKYEEDVNLGSGTRAVVSCAEAPNMKIIGNRIERIRSEHAETWFFDENHP
HS-2015-BA-01   LGRMDGPRRPVKYEEDVNLGSGTRAVVSCAEAPNMKIIGNRIERIRSEHAETWFFDENHP
                ************************************************************

PLCal_ZV        YRTWAYHGSYEAPTQGSASSLINGVVRLLSKPWDVVTGVTGIAMTDTTPYGQQRVFKEKV
HS-2015-BA-01   YRTWAYHGSYEAPTQGSASSLVNGVVRLLSKPWDVVTGVTGIAMTDTTPYGQQRVFKEKV
                *********************:**************************************

PLCal_ZV        DTRVPDPQEGTRQIMSMVSSWLWKELGKHKRPRVCTKEEFINKVRSNAALGAIFEEEKEW
HS-2015-BA-01   DTRVPDPQEGTRQVMSMVSSWLWKELGKHKRPRVCTKEEFINKVRSNAALGAIFEEEKEW
                *************:**********************************************

PLCal_ZV        KTAVEAVNDPRFWALVDKEREHHLRGECQSCVYNMMGKREKKQGEFGKAKGSRAIWYMWL
HS-2015-BA-01   KTAVEAVNDPRFWALVDKEREHHLRGECQSCVYNMMGKREKKQGEFGKAKGSRAIWYMWL
                ************************************************************

PLCal_ZV        GARFLEFEALGFLNEDHWMGRENSGGGVEGLGLQRLGYVLEEMSRIPGGRMYADDTAGWD
HS-2015-BA-01   GARFLEFEALGFLNEDHWMGRENSGGGVEGLGLQRLGYVLEEMSRIPGGRMYADDTAGWD
                ************************************************************

PLCal_ZV        TRISRFDLENEALITNQMEKGHRALALAIIKYTYQNKVVKVLRPAEKGKTVMDIISRQDQ
HS-2015-BA-01   TRISRFDLENEALITNQMEKGHRALALAIIKYTYQNKVVKVLRPAEKGKTVMDIISRQDQ
                ************************************************************

PLCal_ZV        RGSGQVVTYALNTFTNLVVQLIRNMEAEEVLEMQDLWLLRRSEKVTNWLQSNGWDRLKRM
HS-2015-BA-01   RGSGQVVTYALNTFTNLVVQLIRNMEAEEVLEMQDLWLLRRPEKVTNWLQSNGWDRLKRM
                *****************************************.******************

PLCal_ZV        AVSGDDCVVKPIDDRFAHALRFLNDMGKVRKDTQEWKPSTGWDNWEEVPFCSHHFNKLHL
HS-2015-BA-01   AVSGDDCVVKPIDDRFAHALRFLNDMGKVRKDTQEWKPSTGWDNWEEVPFCSHHFNKLHL
                ************************************************************

PLCal_ZV        KDGRSIVVPCRHQDELIGRARVSPGAGWSIRETACLAKSYAQMWQLLYFHRRDLRLMANA
HS-2015-BA-01   KDGRSIVVPCRHQDELIGRARVSPGAGWSIRETACLAKSYAQMWQLLYFHRRDLRLMANA
                ************************************************************

PLCal_ZV        ICSSVPVDWVPTGRTTWSIHGKGEWMTTEDMLVVWNRVWIEENDHMEDKTPVTKWTDIPY
HS-2015-BA-01   ICSSVPVDWVPTGRTTWSIHGKGEWMTTEDMLVVWNRVWIEENDHMEDKTPVTKWTDIPY
                ************************************************************

PLCal_ZV        LGKREDLWCGSLIGHRPRTTWAENIKNTVNMVRRIIGDEEKYMDYLSTQVRYLGEEG---
HS-2015-BA-01   LGKREDLWCGSLIGHRPRTTWAENIKNTVNMVRRIIGDEEKYMDYLSTQVRYLGEEGSTP
                *********************************************************

PLCal_ZV        ---
HS-2015-BA-01   GVL
```

Figure S2: Protein secondary structure comparison between the ZIKV Canadian-imported Thai strain PLCal_ZV and the Brazilian HS-2015-BA-01 strain. Only proteins where aa differences were found are represented. Alpha (α) helix, beta (β) strand and coil are represented as follows:

Alpha Helix 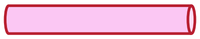

Beta Strand 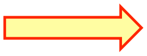

Coil regular sequence

ER anchor for protein C (105-122)

```
PLCal_ZV         GTDTSVGIVGLLLTTAMA
HS-2015-BA-01    GADTSVGIVGLLLTTAMA
                 *:****************
```

Protein prM (123-290)

```
PLCal_ZV         AEVTRRGSAYYMYLDRSDAGEAISFPTTLGMNKCYIQIMDLGHMCDATMSYECPMLDEGV
HS-2015-BA-01    AEVTRRGSAYYMYLDRNDAGEAISFPTTLGMNKCYIQIMDLGHMCDATMSYECPMLDEGV
                 ****************.*******************************************

PLCal_ZV         EPDDVDCWCNTTSTWVVYGTCHHKKGEARRSRRAVTLPSHSTRKLQTRSQTWLESREYTK
HS-2015-BA-01    EPDDVDCWCNTTSTWVVYGTCHHKKGEARRSRRAVTLPSHSTRKLQTRSQTWLESREYTK
                 ************************************************************

PLCal_ZV         HLIRVENWIFRNPGFALAAAAIAWLLGSSTSQKVIYLVMILLIAPAYS
HS-2015-BA-01    HLIRVENWIFRNPGFALAAAAIAWLLGSSTRQKVIYLVMILLIAPAYS
                 ****************************** *****************
```

NS2A (1143-1368)

```
PLCal_ZV         MVTAGSTDHMDHFSLGVLVILLMVQEGLKKRMTTKIIISTSMAVLVAMILGGFSMSDLAK
HS-2015-BA-01    MVTAGSTDHMDHFSLGVLVILLMVQEGLKKRMTTKIIISTSMAVLVAMILGGFSMSDLAK
                 ************************************************************

PLCal_ZV         LAILMGATFAEMNTGGDVAHLALIAAFKVRPALLVSFIFRANWTPRESMLLALASCLLQT
HS-2015-BA-01    LAILMGATFAEMNTGGDVAHLALIAAFKVRPALLVSFIFRANWTPRESMLLALASCLLQT
                 ************************************************************

PLCal_ZV         AISALEGDLMVLINGFALAWLAIRAMVVPRTDNITLAILAALTPLARGTLLVAWRAGLAT
HS-2015-BA-01    VISALEGDLMVLINGFALAWLAIRAMVVPRTDNITLAILAALTPLARGTLLVAWRAGLAT
                 .***********************************************************
```

```
PLCal_ZV          CGGFMLLSLKGKGSVKKNLPFVMALGLTAVRLVDPINVVGLLLLTR

HS-2015-BA-01     CGGFMLLSLKGKGSVKKNLPFVMALGLTAVRLVDPINVVGLLLLTR
                  **********************************************
```

NS3 (1499-2115)

```
PLCal_ZV          TGKRSGALWDVPAPKEVKKGETTDGVYRVMTRRLLGSTQVGVGVMQEGVFHTMWHVTKGS

HS-2015-BA-01     TGKRSGALWDVPAPKEVKKGETTDGVYRVMTRRLLGSTQVGVGVMQEGVFHTMWHVTKGS
                  ************************************************************

PLCal_ZV          ALRSGEGRLDPYWGDVKQDLVSYCGPWKLDAAWDGHSEVQLLAVPPGERARNIQTLPGIF

HS-2015-BA-01     ALRSGEGRLDPYWGDVKQDLVSYCGPWKLDAAWDGHSEVQLLAVPPGERARNIQTLPGIF
                  ************************************************************

PLCal_ZV          KTKDGDIGAVALDYPAGTSGSPILDKCGRVIGLYGNGVVIKNGSYVSAITQGRREEETPV

HS-2015-BA-01     KTKGGDIGAVALDYPAGTSGSPILDKCGRVIGLYGNGVVIKNGSYVSAITQGRREEETPV
                  ***.********************************************************

PLCal_ZV          ECFEPSMLKKKQLTVLDLHPGAGKTRRVLPEIVREAIKTRLRTVILAPTRVVAAEMEEAL

HS-2015-BA-01     ECFEPSMLKKKQLTVLDLHPGAGKTRRVLPEIVREAIKTRLRTVILAPTRVVAAEMEEAL
                  ************************************************************

PLCal_ZV          RGLPVRYMTTAVNVTHSGTEIVDLMCHATFTSRLLQPIRVPNYNLYIMDEAHFTDPSSIA

HS-2015-BA-01     RGLPVRYMTTAVNVTHSGTEIVDLMCHATFTSRLLQPIRVPNYNLYIMDEAHFTDPSSIA
                  ************************************************************

PLCal_ZV          ARGYISTRVEMGEAAAIFMTATPPGTRDAFPDSNSPIMDTEVEVPERAWSSGFDWVTDHS

HS-2015-BA-01     ARGYISTRVEMGEAAAIFMTATPPGTRDAFPDSNSPIMDTEVEVPERAWSSGFDWVTDHS
                  ************************************************************

PLCal_ZV          GKTVWFVPSVRNGNEIAACLTKAGKRVIQLSRKTFETEFQKTKHQEWDFVVTTDISEMGA

HS-2015-BA-01     GKTVWFVPSVRNGNEIAACLTKAGKRVIQLSRKTFETEFQKTKHQEWDFVVTTDISEMGA
                  ************************************************************

PLCal_ZV          NFKADRVIDSRRCLKPVILDGERVILAGPMPVTHASAAQRRGRIGRNPNKPGDEYLYGGG

HS-2015-BA-01     NFKADRVIDSRRCLKPVILDGERVILAGPMPVTHASAAQRRGRIGRNPNKPGDEYLYGGG
                  ************************************************************

PLCal_ZV          CAETDEDHAHWLEARMLLDNIYLQDGLIASLYRPEADKVAAIEGEFKLRTEQRKTFVELM

HS-2015-BA-01     CAETDEDHAHWLEARMLLDNIYLQDGLIASLYRPEADKVAAIEGEFKLRTEQRKTFVELM
                  ************************************************************

PLCal_ZV          KRGDLPVWLAYQVASAGITYTDRRWCFDGTTNNTIMEDSVPAEVWTRYGEKRVLKPRWMD

HS-2015-BA-01     KRGDLPVWLAYQVASAGITYTDRRWCFDGTTNNTIMEDSVPAEVWTRHGEKRVLKPRWMD
                  ***********************************************:************
```

```
PLCal_ZV          ARVCSDHAALKSFKEFA
HS-2015-BA-01     ARVCSDHAALKSFKEFA
                  ****************
```

NS4A (2116-2242)

```
PLCal_ZV          AGKRGAAFGVMEALGTLPGHMTERFQEAIDNLAVLMRAETGSRPYKAAAAQLPETLETIM
HS-2015-BA-01     AGKRGAALGVMEALGTLPGHMTERFQEAIDNLAVLMRAETGSRPYKAAAAQMPETLETIM
                  *******:*******************************************:********

PLCal_ZV          LLGLLGTVSLGIFFVLMRNKGIGKMGFGMVTLGASAWLMWLSEIEPARIACVLIVVFLLL
HS-2015-BA-01     LLGLLGTVSLGIFFVLMRNKGIGKMGFGMVTLGASAWLMWLSEIEPARIACVLIVVFLLL
                  ************************************************************

PLCal_ZV          VVLIPEP
HS-2015-BA-01     VVLIPEP
                  *******
```

NS5 (2517-3417)

```
PLCal_ZV          VKRRGGGTGETLGEKWKARLNQMSALEFYSYKKSGITEVCREEARRALKDGVATGGHAVS
HS-2015-BA-01     VKRRGGGTGETLGEKWKARLNQMSALEFYSYKKSGITEVCREEARRALKDGVATGGHAVS
                  ************************************************************

PLCal_ZV          RGSAKLRWLVERGYLQPYGKVIDLGCGRGGWSYYAATIRKVQEVKGYTKGGPGHEEPMLV
HS-2015-BA-01     RGSAKLRWLVERGYLQPHGKVIDLGCGRGGWSYYAATIRKVQEVKGYTKGGPGHEEPVLV
                  *****************:***************************************:**

PLCal_ZV          QSYGWNIVRLKSGVDVFHMAAEPCDTLLCDIGESSSSPEVEEARTLRVLSMVGDWLEKRP
HS-2015-BA-01     QSYGWNIVRLKSGVDVFHMAAEPCDTLLCDIGESSSSPEVEEARTLRVLSMVGDWLEKRP
                  ************************************************************

PLCal_ZV          GAFCIKVLCPYTSTMMETLERLQRRYGGGLVRVPLSRNSTHEMYWVSGAKSNTIKSVSTT
HS-2015-BA-01     GAFCIKVLCPYTSTMMETLERLQRRYGGGLVRVPLSRNSTHEMYWVSGAKSNTIKSVSTT
                  ************************************************************

PLCal_ZV          SQLLLGRMDGPRRPVKYEEDVNLGSGTRAVVSCAEAPNMKIIGNRIERIRSEHAETWFFD
HS-2015-BA-01     SQLLLGRMDGPRRPVKYEEDVNLGSGTRAVVSCAEAPNMKIIGNRIERIRSEHAETWFFD
                  ************************************************************

PLCal_ZV          ENHPYRTWAYHGSYEAPTQGSASSLINGVVRLLSKPWDVVTGVTGIAMTDTTPYGQQRVF
HS-2015-BA-01     ENHPYRTWAYHGSYEAPTQGSASSLVNGVVRLLSKPWDVVTGVTGIAMTDTTPYGQQRVF
                  *************************:**********************************

PLCal_ZV          KEKVDTRVPDPQEGTRQIMSMVSSWLWKELGKHKRPRVCTKEEFINKVRSNAALGAIFEE
HS-2015-BA-01     KEKVDTRVPDPQEGTRQVMSMVSSWLWKELGKHKRPRVCTKEEFINKVRSNAALGAIFEE
                  *****************:******************************************
```

```
PLCal_ZV        EKEWKTAVEAVNDPRFWALVDKEREHHLRGECQSCVYNMMGKREKKQGEFGKAKGSRAIW
HS-2015-BA-01   EKEWKTAVEAVNDPRFWALVDKEREHHLRGECQSCVYNMMGKREKKQGEFGKAKGSRAIW
                ************************************************************

PLCal_ZV        YMWLGARFLEFEALGFLNEDHWMGRENSGGGVEGLGLQRLGYVLEEMSRIPGGRMYADDT
HS-2015-BA-01   YMWLGARFLEFEALGFLNEDHWMGRENSGGGVEGLGLQRLGYVLEEMSRIPGGRMYADDT
                ************************************************************

PLCal_ZV        AGWDTRISRFDLENEALITNQMEKGHRALALAIIKYTYQNKVVKVLRPAEKGKTVMDIIS
HS-2015-BA-01   AGWDTRISRFDLENEALITNQMEKGHRALALAIIKYTYQNKVVKVLRPAEKGKTVMDIIS
                ************************************************************

PLCal_ZV        RQDQRGSGQVVTYALNTFTNLVVQLIRNMEAEEVLEMQDLWLLRRSEKVTNWLQSNGWDR
HS-2015-BA-01   RQDQRGSGQVVTYALNTFTNLVVQLIRNMEAEEVLEMQDLWLLRRPEKVTNWLQSNGWDR
                *********************************************.**************

PLCal_ZV        LKRMAVSGDDCVVKPIDDRFAHALRFLNDMGKVRKDTQEWKPSTGWDNWEEVPFCSHHFN
HS-2015-BA-01   LKRMAVSGDDCVVKPIDDRFAHALRFLNDMGKVRKDTQEWKPSTGWDNWEEVPFCSHHFN
                ************************************************************

PLCal_ZV        KLHLKDGRSIVVPCRHQDELIGRARVSPGAGWSIRETACLAKSYAQMWQLLYFHRRDLRL
HS-2015-BA-01   KLHLKDGRSIVVPCRHQDELIGRARVSPGAGWSIRETACLAKSYAQMWQLLYFHRRDLRL
                ************************************************************

PLCal_ZV        MANAICSSVPVDWVPTGRTTWSIHGKGEWMTTEDMLVVWNRVWIFENDHMEDKTPVTKWT
HS-2015-BA-01   MANAICSSVPVDWVPTGRTTWSIHGKGEWMTTEDMLVVWNRVWIFENDHMEDKTPVTKWT
                ************************************************************

PLCal_ZV        DIPYLGKREDLWCGSLIGHRPRTTWAENIKNTVNMVRRIIGDEEKYMDYLSTQVRYLGEE
HS-2015-BA-01   DIPYLGKREDLWCGSLIGHRPRTTWAENIKNTVNMVRRIIGDEEKYMDYLSTQVRYLGEE
                ************************************************************

PLCal_ZV        G
HS-2015-BA-01   G
                *
```
